# LC-MS/MS Analysis of Cyanotoxins in Bivalve Mollusks—Method Development, Validation and First Evidence of Occurrence of Nodularin in Mussels (*Mytilus edulis*) and Oysters (*Magallana gigas*) from the West Coast of Sweden

**DOI:** 10.3390/toxins15050329

**Published:** 2023-05-11

**Authors:** Julio César España Amórtegui, Heidi Pekar, Mark Dennis Chico Retrato, Malin Persson, Bengt Karlson, Jonas Bergquist, Aida Zuberovic-Muratovic

**Affiliations:** 1 Science Department, Swedish Food Agency, Box 622, SE-751 26 Uppsala, Sweden; jcespanaa@unal.edu.co (J.C.E.A.); heidi.pekar@svoa.se (H.P.); malin.persson@slv.se (M.P.); 2 Chemistry Department, Science Faculty, Universidad Nacional de Colombia, Cr. 45 N° 26-85, Bogotá P.O. Box 111321, Colombia; 3 Stockholm Water and Waste Company, Bryggerivägen 10, SE-106 36 Stockholm, Sweden; 4 Department of Chemistry, Biomedical Center, Analytical Chemistry and Neurochemistry, Uppsala University, Box 599, SE-751 24 Uppsala, Sweden; mark.retrato@kemi.uu.se (M.D.C.R.); jonas.bergquist@kemi.uu.se (J.B.); 5 Research and Development, Oceanography, Swedish Meteorological and Hydrological Institute, Sven Källfelts Gata 15, SE-426 71 Västra Frölunda, Sweden; bengt.karlson@smhi.se

**Keywords:** LC-MS/MS, cyanotoxins, nodularin, bivalves, mussels, seafood safety

## Abstract

In this paper, an LC-MS/MS method for the simultaneous identification and quantification of cyanotoxins with hydrophilic and lipophilic properties in edible bivalves is presented. The method includes 17 cyanotoxins comprising 13 microcystins (MCs), nodularin (NOD), anatoxin-a (ATX-a), homoanatoxin (h-ATX) and cylindrospermopsin (CYN). A benefit to the presented method is the possibility for the MS detection of MC-LR-[Dha7] and MC-LR-[Asp3] as separately identified and MS-resolved MRM signals, two congeners which were earlier detected together. The performance of the method was evaluated by in-house validation using spiked mussel samples in the quantification range of 3.12–200 µg/kg. The method was found to be linear over the full calibration range for all included cyanotoxins except CYN for which a quadratic regression was used. The method showed limitations for MC-LF (R^2^ = 0.94), MC-LA (R^2^ ≤ 0.98) and MC-LW (R^2^ ≤ 0.98). The recoveries for ATX-a, h-ATX, CYN, NOD, MC-LF and MC-LW were lower than desired (<70%), but stable. Despite the given limitations, the validation results showed that the method was specific and robust for the investigated parameters. The results demonstrate the suitability of the method to be applied as a reliable monitoring tool for the presented group of cyanotoxins, as well as highlight the compromises that need to be included if multi-toxin methods are to be used for the analysis of cyanotoxins with a broader range of chemical properties. Furthermore, the method was used to analyze 13 samples of mussels (*Mytilus edulis*) and oysters (*Magallana gigas*) collected in the 2020–2022 summers along the coast of Bohuslän (Sweden). A complementary qualitative analysis for the presence of cyanotoxins in phytoplankton samples collected from marine waters around southern Sweden was performed with the method. Nodularin was identified in all samples and quantified in bivalve samples in the range of 7–397 µg/kg. Toxins produced by cyanobacteria are not included in the European Union regulatory monitoring of bivalves; thus, the results presented in this study can be useful in providing the basis for future work including cyanotoxins within the frame of regulatory monitoring to increase seafood safety.

## 1. Introduction

Cyanobacteria are photosynthesizing organisms that occur in water and on land, although most cyanobacteria are aquatic and can be found in both marine and freshwater habitats. Traditionally they have been called “blue-green algae” even though not all species are blue-green or even algae. During the mass proliferation of certain planktonic species of cyanobacteria, so called cyanoblooms, a heavy mass of cyanobacteria accumulates in the water and is often visible along the coasts or beaches. The abundant growths of cyanobacteria both in freshwater and coastal ecosystems is associated with increased eutrophication and global climate change which raises serious concerns about harmful algal blooms (HABs) [1]. The harmfulness of cyanoblooms refers to the ability of some cyanobacteria genera to produce secondary metabolites, cyanotoxins, among which the most powerful natural toxic compounds known are represented [2,3,4]. More than 150 genera of cyanobacteria are included in the cyanobacteria class [5] of which an estimated 40 genera account for the production of cyanotoxins [6]; among these are *Microcystis*, *Nodularia*, *Dolichospermum*, *Raphidiopsis raciborskii*, *Aphanizomenon* and *Oscillatoria*. The production of cyanotoxins is not continuous and occurs under certain conditions [7,8,9,10,11,12], particularly upon elevated temperatures and in the presence of high levels of nutrients in the water. When produced, most of cyanotoxins are contained within the cells of cyanobacteria (intracellular) and their release into the surrounding water (free toxins) occurs mostly when the cells die [13].

Microcystins (MCs) occur globally and are the most investigated group of cyanobacterial toxins to which at least 279 analogues belong [8,14,15]. Nodularin is a cyanotoxin mainly produced by *Nodualria Spumigena* typically found in brackish waters [16], of which 10 different structural variants are known [17]. MCs and NODs are cyclic peptides (heptapeptide and pentapeptide, respectively) with a similar chemical structure and a characteristic property containing the amino acid Adda ([(2S,3S,8S,9S)-3-amino-9-methoxy-2,6,8-trimethyl-10-phenyl-4,6-decadienoic acid]) in their molecular structures, which is unique to cyanobacteria [18,19]. Anatoxins (anatoxin-a and homoanatoxin-a, ATX-a and h-ATX) and cylindrospermopsin (CYN) belong to alkaloid cyanotoxins produced by a variety of freshwater cyanobacteria [20,21]. Together with MCs, they are best known to occur in recreational waters or drinking water sources representing an issue for the drinking water producers [22,23,24]. Exposure to cyanotoxins may cause adverse health effects that range from a mild skin rash to serious illness, or in rare circumstances, death [2]. MCs and NOD have a similar mode of action which is hepatotoxic, while anatoxins mimic neurotransmitters and act as neurotoxins. Cylindrospermopsin is a highly polar toxin very stable and persistent in aquatic environments that, unlike other cyanotoxins, is mainly present as a free toxin (extracellular, up to 90%). It is a multi-target alkaloid toxic to liver and kidney tissues and leads to mutagenic and genotoxic effects in human cells [25,26].

As natural products of bacteria that typically inhabit eutrophic water bodies, cyanotoxins pose a risk to animals, domestic as well as wild and marine, and humans [27,28,29,30]. Except through the drinking water, the risk for humans arises upon consumption of aquatic organisms or products that have been contaminated with cyanotoxins such as fish, shellfish or dietary supplements based on cyanobacteria [31,32,33,34], and through other types of food [25].

Only a few guidelines for cyanotoxin levels exist. The World Health Organization (WHO) established a TDI of 0.04 µg/kg body weight for chronic exposure to microcystin–leucine arginine (MC-LR) and recommends a safe limit of 1 µg/L for MC-LR in drinking water [35]. As there are no available guidelines for other cyanotoxin variant concentrations of MC-LR, equivalents are usually used for the determination of all cyanotoxin congeners. Accordingly, a derived value for foodstuffs proposed by the WHO equates to 2.4 µg MC-LR per day for an adult (60 kg), which is taken in the context that most of the available toxicological data are based on a single congener, the lack of knowledge about the effects caused by cyanotoxins mixtures and the potential synergistic effects from other compounds [22]. However, considering the information provided from toxicological studies, local seasonal guidance values have been proposed for exposure from shellfish on a daily basis for several weeks during a bloom period, to prevent cyanotoxin poisoning [36].

It is thus far known that there are about 500 different species of cyanobacteria in Swedish fresh waters, while there are 565 in the Baltic Sea [37,38]. Potentially toxin-producing cyanobacteria have been identified in 45% of the Swedish source waters [23,39], and microcystins are commonly detected in fresh waters while nodularin is commonly found in brackish waters [39,40]. The most relevant toxin-producing cyanobacteria species in the Baltic Sea are *Nodularia spumigena*, *Dolichospermum* sp. and *Aphanizomenon flos-aquae* [41], all of them known as being able to produce cyanotoxins.

Cyanoblooms are rare along the west cost of Sweden [42], the only geographic area where the Swedish commercial bivalve production is located, along the coast of Bohuslän. For the monitoring of diverse algal blooms, including cyanobacteria in seawater samples from this area, light microscopy is used for the identification and enumeration of cells per unit volume of water. The monitoring is conducted complementarily to the regulatory monitoring of marine biotoxins in bivalve mollusks which is performed with chemical methods.

Continuous routine chemical analysis of cyanotoxins in bivalves is not compulsory while counting cyanobacteria cells does not provide any information whether the cells belong to the toxic or non-toxic strain even if they belong to the same species.

In view of the potential risks to human poisoning associated with the consumption of filter feeders, such as mussels and oysters that are able to accumulate cyanotoxins, analysis of these substances in bivalve mollusks is important to maintain shellfish safety.

At present, a regular as well as sufficient scientific basis for the closing of production areas in Sweden is missing or would need to be based on an assumption that the majority of cyanoblooms (~60%) are toxic [24]. Chemical analysis that allows direct confirmation of the presence of multiple cyanotoxin content in bivalve samples is needed as a preferred option.

Various techniques have been applied for the detection of cyanotoxins over the past years. The most common have been enzyme-linked immunosorbent assays (ELISA) [43] and high-performance liquid chromatography (HPLC) with ultraviolet detection (UV) [44,45], although both of these approaches have inherent disadvantages. ELISA is appreciated because it offers high sensitivity, but the known drawbacks of ELISA methods are the incompatibility with organic solvents and the cross reactivity, which in the context of cyanotoxins detection, is caused by the metabolic products of MCs. While such metabolic compounds can cause the overestimation of toxin concentrations in samples, they might not be important to consider from a toxicity perspective [46]. The low sensitivity of UV analysis presupposes that sample purification and concentration steps are introduced before the detection [44,45]. During recent years, mass spectrometry (MS) has, in general, been the most commonly applied approach for the detection of cyanotoxins [18,33,34,47,48,49,50,51,52,53], where ultra-high-performance liquid chromatography (UHPLC) coupled to tandem mass spectrometry (MS/MS) has evolved to become the preferred technique. Among these are methods for analysis of water, fish tissue, algae, algae-based food supplements, fruits and vegetables, and some of the methods have been validated [18,47,49,50]. The contributions using different analytical methodologies in the analysis of cyanotoxins in diverse types of sample matrices have been discussed and summarized in a recent review [54]. This indicates that the risks to human and animal health can potentially be introduced via different routes of exposure. Consequently, there is a desire to develop analytical methods that allow multi-cyanotoxin analysis and at the same time comprise a broad range of sample matrices. However, as for many other natural toxins analyses, the challenge in the development method for analysis of cyanotoxins lies in their inherent diverse physico-chemical properties that defies a uniform analysis of different congener classes without compromises, for instance, in the developing of an optimal multi-cyanotoxin extraction procedure from a complex sample matrix. This usually leads to the priority in method development being given to including toxin congeners with similar properties together such as cyclic peptides MCs with NOD, while the hydrophilic alkaloid cyanotoxins such as ATXs, CYN and saxitoxins (STXs) have a separate approach [55]. Though, in the situations when toxins from both groups are present in a sample collected within a cyanobloom, such an analytical approach will result in some toxins not being detected by the method.

For this study, the priority in using a method that focuses on a defined type of sample yet including a wider range of cyanotoxin congeners belonging to different classes was recognized. Thereby, a broader need for the monitoring of cyanotoxins originating from Swedish marine as well as brackish waters potentially detectable in bivalve samples would be met. To the best of our knowledge, there are no reports on methods utilizing UHPLC-MS/MS which have been developed and validated for the simultaneous extraction and quantification of multi-cyanotoxins with mixed properties, in bivalves. The study focuses only on those cyanotoxin congeners that exist as free toxins in the sample (non-covalently bound in cells) and as such are extractable into methanol, as it has been assumed until now that only free forms are bioavailable [36]. The method includes cyanotoxins that were commercially available at the time of study: ATX-a, h-ATX, CYN, sum of MC-RR [D-Asp3] and MC-RR [D-Asp3, (E)-Dhb7], NOD, MC-LA, MC-LR-[Dha7], MC-LR-[Asp3], MC-LF, MC-LR, MC-LY, MC-HilR, MC-LW, MC-YR, MC-HtyR and MC-WR. The aim was to further evaluate the method performance characteristics and reliability through an in-house validation study. Finally, the method was used to analyze phytoplankton and hypothetically contaminated samples of blue mussel (*Mytilus edulis*) and oyster (*Magallana Gigas*) collected along the coast of Bohuslän during sporadic cyanoblooms in the 2020–2022 time period. The presence of nodularin was confirmed in all samples and for the first time quantified in bivalves from the Swedish west coast.

## 2. Results and Discussion

The blue mussel (*Mytilus edulis*) and oysters (*Magallana Gigas*) inhabit coastal marine waters of Bohuslän and together represent the major part of the bivalve harvesting in Swedish aquaculture industry. This study was planned after an extensive cyanobloom occurred in late summer 2020, which at this time, unusually, also included the Kattegat–Skagerrak area as shown in Supplementary Materials Figure S1, not that far from several mussel and oyster farming areas. A risk of cyanotoxin contamination of these species was hypothesized, but there was no laboratory preparedness with analytical methods that could demonstrate the presence of cyanotoxins in bivalve tissue. Thus, the first aim of the present study was to achieve a robust and straightforward analytical method applicable for the detection and quantification of cyanotoxins in the two representative sample matrices. The assessment of the method performance characteristics and the in-house validation of the method were conducted following, as closely as possible, the guidelines of the EU commission decision 2002/657/EC [56]. The final aim was to analyze samples collected in summer 2020 and in a few sporadic or local cyanobloom events that could threaten the shellfish farming and thereby seafood safety.

### 2.1. Method Development

Cyanotoxins commercially available at the time of conducting the method optimization parameters (ATX, h-ATX, CYN, sum of MC-RR [D-Asp3] and MC-RR [D-Asp3, (E)-Dhb7], NOD, MC-LA, MC-LR [Dha7], MC-LR [Asp3], MC-LF, MC-LR, MC-LY, MC-HilR, MC-LW, MC-YR, MC-HtyR and MC-WR) were included in the current scope. Saxitoxins (STXs), although known as products of cyanobacteria, were excluded from the scope of this study as they are not included in the European Union (EU) regulatory monitoring for paralytic shellfish toxins (PST) [57], as well as other UHPLC-MS/MS-based methods, that have been developed for the analysis of the PST group of toxins in bivalves [58].

**Figure 1:**
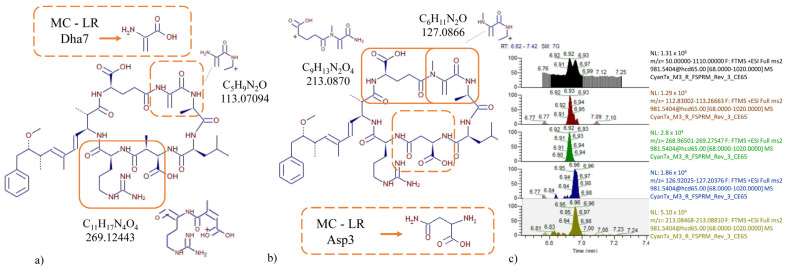
Investigation of selective transitions via LC-HRMS/MS for the toxin pair MC-LR [Asp3] and MC-LR [Dha7]. Structural differences lead to fragments as new candidates for selective transitions. (**a**) MC-LR Dha7 showing the dashed moiety and solid line that yields a unique peak for 113.07094 and 269.12443. (**b**) MC-LR Asp3 showing the solid moieties line that yields unique peaks for 213.0870 and 127.0866. (**c**) Adjacent MS-resolved peaks. The unprecedented, extracted ion chromatograms show excellent S/N ratios in the PRM acquisition mode (CE: 65 eV), which can be readily implemented in an LC-MS/MS (QQQ) for routine analysis.

The separation using liquid chromatography coupled to hybrid quadrupole Orbitrap high-resolution mass spectrometry (LC-HRMS/MS) working in the Parallel Reaction Monitoring (PRM) acquisition mode made it possible to discover selective m/z fragment candidates for the specific detection of two toxins, MC-LR-Asp3 and MC-LR-Dha7, in a single experiment, that were earlier only possible to detect as a pair. Although the transition to the product ion m/z 135 is frequently used for the detection of MCs because it is typically found in high abundance, this transition is not specific enough to selectively detect the critical toxin pair. However, as shown in Figure 1a, a unique fragment containing the dehydroalanine amino acid in the seventh position (Dha7, in dashed lines) is a moiety of particular interest since it yields the fragment C_5_H_9_N_2_O (m/z: 113.07094) which is not present in the MC-LR Asp3 (Figure 1b). This amino acid prevents the formation of a similar but unique fragment C_6_H_11_N_2_O (m/z: 127.0866) shown in the solid small square in Figure 1b. Furthermore, the advantage was also employed for the fragment of C_9_H_13_N_2_O_4_ that is generated in MC-LR Asp3 at m/z: 213.0870 including both the sixth and the seventh position, shown in the larger solid square Figure 1b. On the other hand, the presence of the aspartic acid in the third position is an alternative way to differentiate the two molecules, since the Dha7 yields another private large fragment at this particular position, C_11_H_17_N_4_O_4_ at m/z: 269.12443. The first chromatogram in Figure 1c shows the peaks eluting too close to be determined individually. However, using the above-mentioned m/z values, it is possible to extract the ions from the PRM, and thereby end up with at least two unshared product ions for these isobaric, yet structurally different, molecules.

In order to transfer the new conditions to the triple quadrupole mass spectrometer (QQQ-MS), it was necessary to consider the optimization of the collision energy for the individual selected quantifiers for the transition from the common precursor ion m/z 981.5 in MC-LR [Asp3] and MC-LR [Dha7] to m/z 213 and 269, respectively. Since the precursor undergoes extensive fragmentation that ends up at m/z 135, the collision energy was ramped down in two consecutive tests until an inflexion point was found (Supplementary Materials Figure S2). A collision energy of 55 eV at a constant cone voltage was found to outperform the predecessor common transition towards m/z 135 in terms of selectivity, while keeping adequate sensitivity for the detection needs.

**Figure 2:**
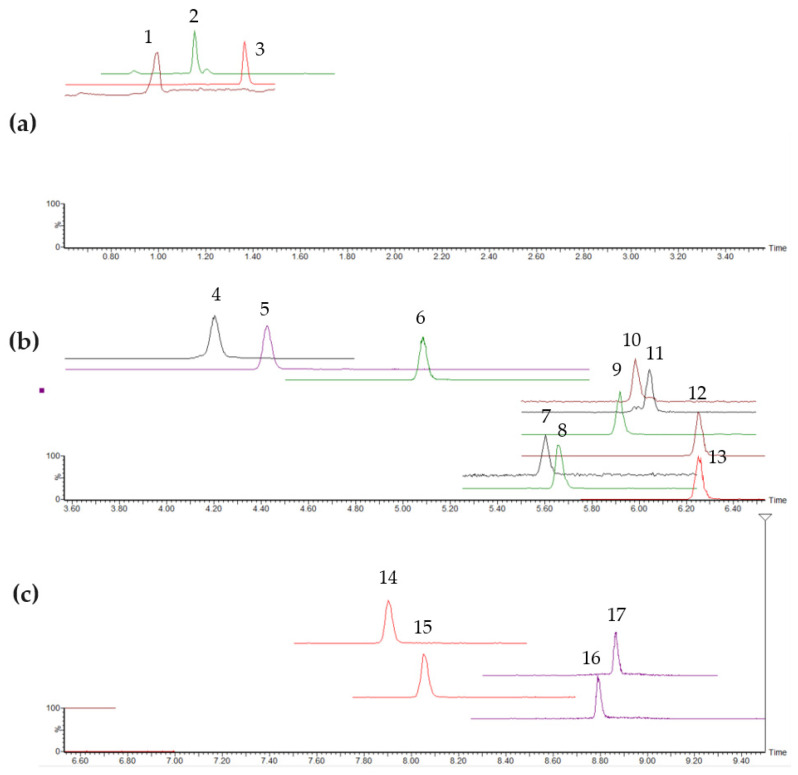
MRM chromatograms of matrix-matched calibration standard of cyanotoxins at the LOQ level by LC-MS/MS, split in sections of early-, middle- and late-eluting compounds. (**a**) Early-eluting compounds: 1. cylindrospermopsin, 2. anatoxin-a and 3. homoanatoxin-a. (**b**) Middle-eluting compounds: 4. microcystins RR [D-Asp3] and microcystins RR [D-Asp3, can-Dhb7] isobaric pair, 5. microcystin RR, 6. nodularin, 7. microcystin YR, 8. microcystin HtyR, 9. microcystin LR, 10. microcystin LR [Dha7], 11. microcystin LR [Asp3], 12. microcystin HilR and 13. microcystin WR. (**c**) Late-eluting compounds: 14. microcystin LA, 15. microcystin LY, 16. microcystin LW and 17. microcystin LF.

The optimization of gradient parameters resulted in the acceptable separation of 17 toxins achieved within 9 min of using an 11 min gradient (Figure 2). Several protocols for the extraction of toxins spiked in bivalve tissue were tested using mixtures of MeOH and water in different proportions, with and without week acidification with acetic acid that did not improve the extraction efficiency [18]. The best recoveries of individual toxins were obtained when pure MeOH was used in a single step extraction and subsequent dilution with Milli-Q water in proportions 1:4 (methanolic sample extract:water). Thus, for the final extraction protocol, 4 mL of MeOH (100%) was chosen as solvent to extract cyanotoxins from 2 g of homogenized tissue, following 2 min of shaking on vortex. The complete extraction protocol used throughout the study is presented as a flow chart in Supplementary Materials Figure S3.

### 2.2. Method Validation

#### 2.2.1. Specificity

To investigate the specificity of the method, individual shellfish samples were used, each corresponding to a homogenate of at least 15 individual mussels harvested at a specific location and time. The samples were analyzed over three different days for the presence of peaks that could disturb or be misidentified as peaks of the cyanotoxins included in the method. For most of the cyanotoxin analytes, no major interferences were observed in chromatograms for the supposedly blank samples. Although, the first and the last parts of the chromatogram were subjected to a closer examination. The presence of a peak with a significant S/N ratio in the vicinity of ATX-a, that was an adjacent baseline-resolved peak (Supplementary Material Figure S4a), may correspond to phenylalanine as it has been described elsewhere [59]. On the contrary, this was not the case with h-ATX (Supplementary Material Figure S4b), since the presence of the peak at the same retention time in the blank sample was evident although with a peak height below 10%, relative to the peak area in the lowest calibration level. The corresponding concentration of 0.39 ng/mL (equivalent to 3.13 µg/kg) was well below the defined LOQ (12.5 µg/kg). Due to the matrix-generated background, an acceptable S/N ratio for CYN was achieved at the level of 0.78 ng/mL (equivalent to 6.25 µg/kg). In a similar way, the presence of a matrix interference over the sum peak of microcystins RR [D-Asp3] + RR [D-Asp3, (E)-Dhb7] in blank was adding up a shoulder to the signal at the lowest concentration tested (Supplementary Material Figure S5a,b). In order to overcome this, a set of alternative transitions were tested but none of them were selective enough (Supplementary Material Figure S5c). However, the use of HRMS in PRM acquisition mode (17500 FWHM @ 200 m/z) enabled a confirmation that the peak in the blank was not the compound of interest by clearing up the interference from the signal of the product ion at m/z 135.08023, along with the protonated precursor (Supplementary Material Figure S5d). Consequently, to ensure that the toxin peak prevailed over the interference on MC-RR [D-Asp3] + RR [D-Asp3, (E)-Dhb7] (Figure S4d), the LOQ was safely set at 6.25 µg/kg. As a result, the interference was only seen as a small shoulder (<30%).

For the other toxins, no interferences from the matrix components were found at the lowest calibrated level, in any of the samples used for the selectivity assessment, that could affect the detection and quantitation of cyanotoxins (blank samples not shown). The extracted ion chromatograms for the MRM transitions in Figure 2 show the selective detection of each cyanotoxin analogue, including the novel detection of MC-LR [Dha7] and MC-LR [Asp3] as separately identified and MS-resolved MRM signals in a calibration standard in a matrix at the LOQ level. Finally, when analyzing the chromatograms of a series of solvent blanks injected after the calibration solution with the highest cyanotoxin concentration, no cross-contamination was observed, ensuring that an analysis of a programmed sample sequence without carry-over effect of any of the cyanotoxins could be performed.

#### 2.2.2. Calibration, Linearity and Matrix Effects in ESI-MS

Two series of calibration standard solutions were analyzed (in solvent and matrix-matched) to assess matrix effects. The matrix-matched calibration curve was analyzed in four set-ups on each of three different days to evaluate the linearity of each of the toxins. The calibration standard solutions were prepared at seven concentration levels in each of the calibration series. The derived calibration curves showed that a linear-fit model was the preferred model in the tested calibration range for both calibration series and for all toxins except CYN, for which a quadratic regression fitted best. The weight of the regression for each toxin was chosen based on the standard deviation of the residuals for optimal precision. The two calibration series were used to assess the matrix-related signal effects in the electrospray ionization indicating suppression or enhancement of the signal if below or above 100%, respectively. The results of the regression and the matrix effects are presented in Table 1 and demonstrate the importance of using a matrix-matched calibration curve in the quantitative analysis of cyanotoxins in authentic bivalve samples. The regression for each toxin in a matrix was generally acceptable, as evidenced by the correlation coefficients with the majority ≥0.98, except for the MC-LF with the R^2^ = 0.94. Due to its poorer linearity performance, an alternative could be to apply a semi-quantitative approach for MC-LF. The linearity parameters with R^2^ ≤ 0.98 need to be considered a necessary compromise even if lower than typically recommended (>0.99) in order to keep the wide toxin scope of harmful congeners within the multi-method. This highlights the importance of including toxins for which the validation data do not completely fulfill the strict validation criteria, if new multi-methods for the analysis of toxins with a broader range of chemical properties are to be achievable. The summary of all the results following the assessments of linearity and matrix effects is shown in Table 1.

#### 2.2.3. Limit of Quantification

The entire calibration range presented in Table 1 expressed in ng/mL was composed of the method validation. Conversion from ng/mL to µg/kg, a unit more convenient to apply in the expression of toxin content per kilogram of raw mussel flesh, is achieved by multiplying by a factor of 8. The LOQs for cyanotoxins were then experimentally determined in individual, spiked mussel tissue homogenates in nine replicates over three batches of analysis. The criteria for signal-to-noise ratio (S/N) were set to ≥10 for the evaluation of the LOQs. The LOQs expressed in µg/kg ranged between 6.29 and 12.11 equating to concentrations of 0.79 and 1.51 ng/mL. Overall, the method was found to show a good performance in the determination of the cyanotoxins in LC-MS/MS at the chosen concentration levels and the results from the LOQ determination are summarized in Table 2.

#### 2.2.4. Recovery

Estimation of the method accuracy was conducted through repeat analysis of spiked blank samples as no appropriate certified reference material (CRM) with known content of cyanotoxins (uncertainty level) was commercially available at the time of this study. Recoveries of cyanotoxins were determined through analysis of nine individually spiked mussel samples at 50, 100 and 200 µg/kg. The crude extracts were diluted with Milli-Q water (1:4), filtered and analyzed by LC-MS/MS on three separate days. The recovery values presented in Table 3 were calculated from peak areas of measured values and the spiked (nominal) values. Most individual toxin recoveries ranged from 70 to 82%, although recoveries were notably lower for ATX-a, h-ATX, microcystin-LF/LW ranging from 51–61% and CYN 19%. Lower recoveries were expected for these toxins due to their different physico-chemical properties. Although the recoveries were lower for some toxins, as long as the precision is acceptable, a recovery correction factor can be applied for toxin content determination, if needed, since there are currently no recommendations for the presence of cyanotoxins in foods. In previous studies, the recovery of MC-LR-[Dha7] and MC-LR-[Asp3] has not been presented in the analysis of cyanotoxins in bivalves due to the inability to resolve these two congeners as separate MS/MS signals. In this work, the recoveries of MC-LR-[Dha7] and MC-LR-[Asp3] were 79 and 70%, respectively, which was expected and in good coherence with the recoveries of the MC congeners with similar properties. The values obtained so far will be observed, and the measures to increase the recoveries for the low-recovering toxins will be conducted continuously to improve their recovery performance. In general, the values of relative standard deviations in repeatability of the recovery within and between batches, shown in Table 3, are acceptable.

#### 2.2.5. Results from Qualitative and Quantitative LC-MS/MS Analysis of Cyanotoxins in Field Samples

##### Analysis of Phytoplankton

As the Baltic Sea phytoplankton samples from late summer have been reported to contain a mixture of cyanobacteria (*N. spumigena*, *Aphanizomenon* sp. and *Anabaena* sp.) [60], a range of cyanotoxin congeners could be expected to detect in the phytoplankton samples collected for this study. Targeted analysis of the entire range of cyanotoxins comprising the validated method was therefore reasonable to perform for all the phytoplankton samples in order to predict which cyanotoxins could be detectable in the bivalve samples.

Fourteen samples of phytoplankton were analyzed for the presence of cyanotoxins. Since the validation method did not comprise assessment of the phytoplankton, as a primary sample matrix, estimated concentrations for the only identified toxin, nodularin, are shown in Table 4. The results from these analyses are in coherence with the earlier published reports on nodularin as the most abundant peptide toxin in the Baltic Sea, produced by cyanobacterium *Nodularia spumigena*, typically present both in cyanoblooms and in the water surrounding the cyanobacteria [42,60].

##### Analysis of Cyanotoxins in Authentic Bivalve Samples

Cyanotoxin-contaminated bivalve samples from 13 different locations along the coast of Bohuslän were used, 10 blue mussel and 3 oyster samples. The cyanotoxins in the samples were considered as identified and quantified when LOQ levels of each toxin were exceeded. The quantitative results from the analysis of bivalve samples, shown in Table 5, confirmed the presence of nodularin as the only toxin. No signals for any of the other cyanotoxins comprising this method could be identified that fit the MRM ratio and the retention time of the reference standards solution, neither above nor below their LOQs down to the lowest calibration solution corresponding to a S/N ratio ≥ 3. Interestingly, nodularin was thereby identified for the first time in bivalve samples from the Swedish west coast. Most of the samples obtained in 2020, in the time period when the presence of cyanobacteria in the sea along the west coast was obvious (Supplementary Materials Figure S1), contained nodularin in a range between 11 and 33 µg/kg. One sample contained 142.3 µg/kg of nodularin and was, unlike the other samples which were blue mussels, the only oyster sample obtained this summer. In samples from 2021, generally, lower levels of nodularin were found compared to samples from 2020, and again higher levels of nodularin were found in the oyster sample. One oyster sample from a local outbreak of *Nodularia spumigena* in September 2022 contained a significantly higher level of nodularin compared to the other samples in this study. The high nodularin concentration found in this sample correlated well with the expectations of its analysis due to the high level of *Nodularia spumigena* measured in the sea water sample from the same area a few days before (15,239 100 µm-lengths/L). Since there is still no regulatory guideline established within the EU for how much nodularin can be present in shellfish aimed for human consumption to ensure that the shellfish is safe, the interpretation of the found nodularin levels in this study remains a challenge. In several non-European countries, local guidelines and limit values are followed for various food matrices such as fish and bivalve mollusks and for various cyanotoxin congeners, including MCs, NOD, CYN and ATX, although these guidelines and limits are applied in different ways [61]. However, such guidelines could serve as references for extrapolation in the interpretation of the nodularin levels found in our study, or in the approaches to shellfish safety. Moreover, it was not obvious why samples of *Magallana gigas* contained higher levels of nodularin, but it could be that this species tends to accumulate nodularin to a greater extent than *Mytilus edulis*, or that the habitat of *Magallana gigas* entails a higher exposure to nodularin. Nevertheless, as this is the first report on the presence of a cyanotoxin in bivalves from the Swedish west coast, further studies are needed to increase the knowledge of species-related nodularin accumulation and provides clear evidence of a possible cyanotoxin profile along the marine coastline where the bivalve mollusks for human consumption are harvested.

## 3. Conclusions

Due to climate changes that lead to changes in the dynamics of ecosystems of water habitats, the determination of emerging cyanotoxins in bivalves is becoming of equally high importance as regulated marine biotoxins to protect human health. A robust and easy-to-use method has been developed enabling the simultaneous determination of anatoxins, cylindrospermopsin, nodularin and microcystins in bivalve tissue with LC-MS/MS. The method is aimed at providing an early warning tool for the Swedish shellfish production to more easily face potential future challenges with harmful cyanoblooms affecting shellfish farming and thereby to increase the shellfish safety. The method has good performance, as shown in the evaluation of specificity, linearity, reproducibility and accuracy. In addition, the method was used to analyze field samples of phytoplankton and bivalves suspected of accumulating cyanotoxins during sporadic cyanoblooms in the 2020–2022 summers. The presence of nodularin was confirmed in all samples and quantified in bivalve samples. This is the first report on cyanotoxin nodularin presence in bivalves from the west coast of Sweden which was directly related to temporal bloom presence. Future work will be focused on extending the scope of the method to include more cyanotoxins.

## 4. Material and Methods

### 4.1. Chemicals, Consumables and Standards

Solvents used for mobile phase preparation and all other chemicals were of LC-MS grade where possible: acetonitrile (ACN, Fisher Scientific, Loughborough, UK), methanol, and formic acid 98–100% (Merck, Darmstadt, Germany). LC–MS grade water was produced by a Milli-Q purification system (Millipore, Solna, Sweden). Discardit II 10 mL polypropylene syringes were from Beckton, Dickinson and Company (Franklin Lakes, NJ, USA), PVDF filters 0.22 µm, Ø 13 mm from Whatman (Maidstone, UK) and glass fibre filters (GF/F) pore size 0. 22 µm, Ø 47 mm were from Millipore (Merck Life Science, Solna, Sweden). The reference standards of cyanotoxins included in this method (anatoxins, cylindrospermopsin, nodularin and microcystins) were ordered from several sources (Supplementary Materials Table S1). When possible, standards were purchased as solutions, while some of the substances were only available in solid form. Stock solutions of 5000 µg/L in methanol were therefore prepared in-house. All stock solutions were stored in darkness at −20 °C. Three separate mixtures were prepared with toxins divided between the mixtures and each toxin’s concentration was 625 µg/L in each solution (Supplementary Materials Table S1). The three standard solution mixtures were pooled (1:1:1, *v*/*v*) to achieve a working standard solution with a concentration of 208.3 µg/L for each toxin. The matrix-matched calibration standards were prepared through a serial dilution as follows: 24 µL of the working solution was diluted with 76 µL of Milli-Q water, where 50 µL of the diluted solution was then transferred to the next vial before 50 µL of blank mussel extract (previously diluted 1:1 in Milli-Q water) was added, to give the calibration solution with the highest toxin concentration (25 µg/L). In the next vial, containing 50 µL from the previous step, 50 µL of Milli-Q water was added where after 50 µL of the mixture was transferred to the next vial before 50 µL of blank extract was added to give the calibration solution of 12.5 µg/L, etc. The calibration points were 25.00, 12.50, 6.25, 3.13, 1.56, 0.78 and 0.39 µg/L; this method can be expressed in µg/kg of bivalve flesh by multiplying by a factor of 8.

### 4.2. Samples and Sample Preparation

#### 4.2.1. Bivalve Samples

Fresh blue mussels (*Mytilus edulis*) and oysters (*Magallana gigas*) for the validation work were purchased from local grocery stores. The shellfish were shucked to remove the flesh from the shells before homogenization to form a smooth and lump-free slurry. An additional seven samples, presumably free of cyanotoxins within the present scope, used for selectivity evaluation were obtained from different shellfish farming areas at the time where no algae or cyanoblooms were in evidence. In general, at least 15 whole individuals without shells were used for one sample (approximately 50–200 g). The homogenized tissue was weighted into 50 mL centrifuge tubes in aliquots (2.0 g) and stored in −20 °C until used as blank matrices or for spiking experiments. In addition to the blank materials, hypothetically positive bivalves (*Mytilus edilus* and *Magallana gigas*) were collected from different sites within shellfish farming areas during July or August in 2020 and 2021. One sample (*Magallana gigas*) was obtained upon a local outbreak of *Nodularia Spumigena* in the vicinity of an oyster production area in September 2022. These samples were shucked to remove the shells from the flesh that was stored in −20 °C until homogenization prior to the extraction and analysis using the validated LC-MS/MS method.

#### 4.2.2. Phytoplankton Samples

Phytoplankton samples were collected from a research vessel during an annual environmental monitoring tour around southern Sweden (Supplementary Material, Figure S6). The geographical vicinity and timing for the conduction of the phytoplankton sampling was as close as it was possible to the sampling of bivalves, in July 2021 (sampling dates available in Table 4 and Table 5). Phytoplankton samples were either collected on a Millipore filter using a hose to filter 400–500 mL of sea water through the filter or by collecting the biomass of the cyanobloom from the water surface, where visible, into 50 mL centrifuge tubes. After the completed filtration, each filter with phytoplankton material was folded, placed in cryo tubes and stored at −20 °C. Before the LC-MS/MS analysis, all the phytoplankton samples (on filter in cryo tubes and in centrifuge tubes) were subjected to a freeze–thaw procedure to extract intracellular cyanotoxins as follows: the samples were placed in a freezer at −80 °C, removed from the freezer after 20 min and thawed in a room temperate water bath. The freeze–thaw procedure was repeated twice. Each filter with phytoplankton was cut into 3–4 strips, inserted into its original cryo tube and 400 µL of MeOH (100%) was added. To each centrifuge tube containing cyanobloom biomass, MeOH was added in a volume corresponding to the volume of the biomass. All samples were vortexed for 2 min, shaken for 20 min on a shaking table, filtered using a 0.22 µM Whatman filter with a syringe and diluted 1:4 with Milli-Q water (sample filtrate: Milli-Q water, *v*/*v*) before analysis with LC-MS/MS.

### 4.3. LC-MS/MS (QQQ)

Chromatography was conducted using an ACQUITY I-Class UPLC system (Waters, Manchester, UK). Separation was achieved with an ACQUITY BEH C_18_ UPLC column (P/N 186002352, Lot no. 0385310401), 2.1 mm × 100 mm fitted with a VanGuard ACQUITY BEH C_18_ UPLC pre-column (P/N 186003975, lot no. 0387310531), 2.1 mm × 5 mm, both having a particle size of 1.7 µm (Waters, Manchester, UK). The columns were held at +60 °C during analysis, with samples held in the sample manager at +15 °C, and injection volume was 5 µL. Mobile phase A was 0.025%/*v*/*v*) formic acid (FA) in Milli-Q water and mobile phase B was 0.025% (*v*/*v*) formic acid (FA) in acetonitrile (ACN). The gradient elution was performed at 0.4 mL/min as follows: 0–1.0 min, 2–25% B; 1.0–3.0 min, 25% B; 3.0–6.0 min, 25–40% B; 6.0–8.0 min, 40–50% B; 8.0–8.2 min, 50–95% B, 8.2–9.0 min, 95% B; 9.0–10.0 min, 95–2% B; 10.0–11.0, 2% B. Quantification was performed in multiple reaction monitoring (MRM) mode using a triple quadrupole mass spectrometer (MS/MS), Xevo TQ-S from Waters (Manchester, UK). The mass spectrometer was used in positive electrospray mode (ES+), with a capillary voltage of 1.0 kV. The source offset was 50 V and the source temperature was 150 °C. Nitrogen (N_2_) was used as desolvation and cone gas at flows of 600 and 150 L/h, respectively. The desolvation gas temperature was 600 °C. The nebulizing gas, also N_2_, was at a pressure of 7.0 bars. Argon (Alphagas, Malmö, Sweden) was used as collision gas at a flow of 0.15 mL/min. The compound-specific mass spectrometric parameters such as cone voltage (CV), collision energy (CE) and m/z transitions are summarized in Supplementary Materials Table S2 [62,63].

### 4.4. LC-HRMS/MS (Hybrid Q-Orbitrap)

Chromatographic separation followed by mass spectrometry analysis was per-formed in an UHPLC Dionex^TM^ Ultimate 3000 (Thermo Scientific^TM^, San Jose, CA, USA) coupled to a Q Exactive^TM^ Focus mass spectrometer (Thermo Scientific^TM^, Bremen, Germany). Separation was achieved with an Accucore aQ UHPLC C18 stationary phase column (Thermo Scientific™, San Jose, CA, USA) of length, diameter and particle size of 100 mm, 2.1 mm and 2.6 µm, respectively. The column was held at +60 °C during analysis, with samples held in the sample manager at +8 °C, and injection volume was 10 µL. The mobile phases and the gradient ramp were the same as described for the LC-MS/MS (QQQ). However, the HRMS/MS (Hybrid Q-Orbitrap) was equipped with a heated electrospray ionization source (HESI II). Optimal conditions were the following: spray voltage of 3.5 kV (positive and negative mode), sheath gas flow rate of 35 psi, auxiliary gas flow rate of 12 arbitrary units (au), sweep gas flow rate of 0 au, capillary temperature of 210 °C, auxiliary gas temperature of 350 °C and S-lens radiofrequency of 50. Two acquisition modes were used for the analysis, full scan and PRM. The conditions for the FS (MS1) were resolving power (RP) of 70,000 FWHM, Automatic Gain Control (AGC) of 1 × 10^6^ charges, Maximum Injection Time (maxIT) set to automatic in a preliminary stage to find the toxins via the exact mass of the protonated adducts with an extraction window of 5 ppm. For the PRM method, the isolation windows were set at 3 Da, resolving power (RP) of 17,500 FWHM, Automatic Gain Control (AGC) of 5 × 10^5^ charges, Maximum Injection Time (maxIT) and CE of 25 eV. The inclusion list used for the specific mass spectrometric accurate mass is presented in Supplementary Materials Table S3. An external mass calibration was performed daily during the experiments with a mix of n-butylamine, caffeine, Ultramark 1621 and MRFA. The data analysis was performed using the software TraceFinder v4.0 (Thermo Scientific™, San Jose, CA, USA).

## Figures and Tables

**Table 1:** Matrix-matched calibration curves for 17 different cyanotoxins, including 13 MCs, NOD, ATX-a, h-ATX and CYN. Matrix effect was evaluated as %ME for each toxin: %ME < 100—signal suppression, %ME > 100—signal enhancement. * Weighted least squares regression. ** *Italic* font style represents the calibration range (Cal. Range) for the isobaric toxin pair reported as sum of Microcystins RR [D-Asp3] + Microcystins RR [D-Ascan (E)-Dhb7].

Toxins	Cal. Range	Regression			%ME
	(ng/mL)	Type	WLS *	r^2^	Mussel
Anatoxin A	0.39–25	Linear	1/X^2^	0.998	78
Homoanatoxin A	0.39–25	Linear	1/X^2^	0.998	80
Cylindrospermopsin	0.78–25	Quadratic	1/Y	0.988	16
Microcystins RR Sum **	*0.78–25*	*Linear*	*1/X*	*0.993*	*115*
Microcystin RR	0.39–25	Linear	1/X	0.992	107
Nodularin	0.39–25	Linear	1/X	0.998	104
Microcystin LA	0.39–25	Linear	1/Y	0.977	125
Microcystin LR [Dha7]	0.78–25	Linear	1/X	0.991	171
Microcystin LR [Asp3]	0.78–25	Linear	1/X	0.992	162
Microcystin LF	0.39–25	Linear	1/X^2^	0.944	61
Microcystin LR	0.39–25	Linear	1/X	0.992	167
Microcystin LY	0.39–25	Linear	1/Y	0.989	118
Microcystin HilR	0.39–25	Linear	1/X	0.987	175
Microcystin LW	0.78–25	Linear	1/X^2^	0.978	82
Microcystin YR	0.39–25	Linear	1/X	0.992	208
Microcystin HtyR	0.39–25	Linear	1/X	0.990	181
Microcystin WR	0.78–25	Linear	1/X	0.984	179

**Table 2:** Validated limits of quantification (LOQs) of each cyanotoxin congener and the recovery percentages for within and between-batch repeatabilities. RSDr: within-batch repeatability; RSD_R_: between-batch repeatability. * All values are based on three repetitions on different days, n = 9. ** *Italic* values represent isobaric pair reported as a sum of Microcystins RR [D-Asp3] + Microcystins RR [Dcanp3, (E)-Dhb7].

Toxins	Calculated (ng/mL)	LOD * (µg/kg)	LOQ * (µg/kg)	Recovery (%)	RSD_r_ (%)	RSD_R_ (%)
Anatoxin A	0.94	2.51	7.52	60.3	1.6	6.6
Homoanatoxin A	0.81	2.17	6.50	52.1	1.6	6.9
Cylindrospermopsin	1.16	3.10	9.30	18.6	7.4	15.0
Microcystins RR Sum **	1.41	3.75	11.26	90.3	1.5	5.6
Microcystin RR	1.48	3.95	11.85	94.9	1.3	7.6
Nodularin	0.98	2.61	7.83	62.7	2.4	7.1
Microcystin LA	1.09	2.92	8.76	70.2	2.4	8.6
Microcystin LR [Dha7]	1.35	3.59	10.78	86.4	3.0	9.4
Microcystin LR [Asp3]	1.16	3.10	9.29	74.4	3.0	11.9
Microcystin LF	0.79	2.10	6.29	50.4	5.0	18.0
Microcystin LR	1.22	3.25	9.74	78.1	1.7	6.6
Microcystin LY	1.19	3.17	9.50	76.1	1.9	7.8
Microcystin HilR	1.32	3.52	10.55	84.5	1.6	6.3
Microcystin LW	0.96	2.57	7.70	61.7	3.4	12.3
Microcystin YR	1.32	3.53	10.58	84.7	5.5	16.9
Microcystin HtyR	1.15	3.07	9.21	73.8	1.6	6.4
Microcystin WR	1.51	4.04	12.11	97.0	1.6	6.4

**Table 3:** Matrix-matched calibration curves for different types of cyanotoxins. Recovery percentages (Rec. %) for within- and between-batch repeatabilities. RSDr: within-batch repeatability (n = 9). RSD_R_: between-batch repeatability (n = 18). All values are based on three repetitions on three different days, n = 9. ** *Italic* values represent isobaric pair reported as a sum of Microcystins RR [D-Asp3] + Microcystins RRcan-Asp3, (E)-Dhb7].

Toxins	Low-Level Spike (50 µg/kg)			Mid-Level Spike (100 µg/kg)			High-Level Spike (200 µg/kg)		
	Rec. (%)	RSD_r_ (%)	RSD_R_ (%)	Rec. (%)	RSD_r_ (%)	RSD_R_ (%)	Rec. (%)	RSD_r_ (%)	RSD_R_ (%)
Anatoxin A	60.6	1.6	4.1	57.9	1.8	6.0	58.9	1.2	12.4
Homoanatoxin A	52.2	1.5	4.5	48.6	1.7	5.2	54.0	2.1	8.4
Cylindrospermopsin	18.6	7.4	15.0	19.1	4.7	11.3	30.7	4.5	13.5
Microcystins RR Sum **	72.4	2.8	6.0	66.1	1.7	5.4	70.5	2.0	6.7
Microcystin RR	81.6	2.8	13.6	71.5	1.7	7.1	79.8	2.1	5.1
Nodularin	63.5	2.2	4.4	62.3	1.6	6.1	74.2	1.7	6.7
Microcystin LA	69.5	3.1	12.3	65.6	1.4	7.5	75.3	3.0	27.7
Microcystin LR [Dha7]	78.7	3.0	8.9	74.6	1.7	5.1	88.7	2.8	9.3
Microcystin LR [Asp3]	70.3	2.8	8.4	67.3	2.3	6.7	82.5	2.5	9.9
Microcystin LF	51.4	2.7	7.5	49.2	4.3	10.7	83.0	7.3	25.5
Microcystin LR	75.2	3.4	7.8	72.4	2.7	6.6	86.2	2.0	7.9
Microcystin LY	68.9	1.6	4.4	65.5	1.1	6.4	73.5	2.6	11.9
Microcystin HilR	77.9	2.9	6.7	76.0	2.3	6.5	87.4	1.7	7.3
Microcystin LW	53.2	1.5	7.2	50.6	1.9	7.8	72.2	5.2	17.9
Microcystin YR	77.6	3.4	10.9	71.6	1.8	5.5	88.8	2.9	9.9
Microcystin HtyR	71.5	3.8	8.8	69.5	2.0	6.7	82.9	2.9	8.6
Microcystin WR	81.7	3.3	7.6	76.8	1.4	5.2	87.2	2.0	6.3

**Table 4:** Qualitative analysis of phytoplankton samples. ND, non-defined amount of phytoplankton biomass analyzed. * Estimated quantity (µg/L) in extract from freeze–thawed samples. ** The quantity of nodularin far beyond the calibration range.

Sample Identity	Sampling Station	Date	Time	Filtered Volume (mL)	Nodularin * (µg/L)
SLV-01, Släggö, HOSE, 20210713	Släggö	2021-07-13	07:00	500	<LOQ
SLV-02, Å17, HOSE, 20210713	Å17	2021-07-13	15:45	500	<LOQ
SLV-03, N14, HOSE, 20210714	N14	2021-07-14	05:05	500	8.4
SLV-04, Anholt, HOSE, 20210714	Anholt	2021-07-14	08:00	500	6.1
SLV-05, BY2, HOSE, 20210715	BY2	2021-07-15	05:15	500	10.8
SLV-06, BY5, HOSE, 20210715,	BY5	2021-07-15	14:45	400	5.7
SLV-07, BY15, HOSE, 20210716	BY15	2021-07-16	18:00	400	12.4
SLV-08, BY38, HOSE, 20210717	BY38	2021-07-17	11:30	500	16.2
SLV-09, RefM1V1, HOSE, 20210717	RefM1V1	2021-07-17	23:20	400	26.1
SLV-10, BY4, SURFACE, 20210715	BY4	2021-07-15	11:20	ND	**
SLV-11, BY5, SURFACE, 20210715	BY5	2021-07-15	14:45	ND	**
SLV-12, BY10, SURFACE, 20210716	BY10	2021-07-16	09:55	ND	**
SLV-13, BY39, SURFACE, 20210717	BY39	2021-07-17	20:00	ND	**
SLV-14, Hanö, SURFACE, 20210718	Hanö Bight	2021-07-18	06:30	ND	**

**Table 5:** Quantitative analysis of cyanotoxins in bivalve samples. Two aliquots of each sample were analyzed. w: week, DD/MM-YY, MG: *Magallana gigas*, ME: *Mytilus edulis*. * Ion ratio for the product ions 825.5 > 135 and 825. 5 > 103 of all the findings was ±20% of the average ratio of all the levels used in the matrix-matched calibration curve.

Sample Name	Date	Sampled Species	Ion Ratio Duplicate *		Nodularin (µg/kg)	Sample Duplicate Diff. (%)
			Sample a	Sample b		
SLV-1, Cyano	w32, 02/8-20	MG	1.72	1.65	142.3	1.1
SLV-2, Cyano	w33, 10/8-20	ME	1.66	1.57	28.8	0.1
SLV-3, Cyano	w33, 10/8-20	ME	1.59	1.59	11.4	4.7
SLV-4, Cyano	w33, 10/8-20	ME	1.64	1.59	27.3	0.5
SLV-5, Cyano	w33, 09/8-20	ME	1.60	1.63	26.7	0.9
SLV-6, Cyano	w34, 17/8-20	ME	1.63	1.57	31.2	5.1
SLV-7, Cyano	w34, 19/8-20	ME	1.60	1.60	26.0	0.1
SLV-8, Cyano	w34, 16/8-20	ME	1.58	1.60	33.1	3.4
SLV-9, Cyano	w28, 13/7-21	ME	1.65	1.55	6.7	1.1
SLV-10, Cyano	w28, 13/7-21	ME	1.58	1.55	7.6	4.0
SLV-11, Cyano	w28, 18/7-21	ME	1.55	1.59	10.9	1.8
SLV-12, Cyano	w28, 18/7-21	MG	1.53	1.61	24.1	0.6
SLV-13, Cyano	w37, 18/9-22	MG	1.67	1.63	397.3	1.2

## Data Availability

Not applicable.

## Supplementary Materials

The following supporting information can be downloaded at: https://www.mdpi.com/article/10.3390/toxins15050329/s1, Figure S1: Cyanobacteria bloom; Figure S2: Optimization of the collision energy (eV) carried out in two subsequent MRM tests including the ranges in search for an inflexion point. Figure S3: Protocol for the extraction of cyanotoxins from bivalve tissue. Figure S4: Selectivity in mussel matrix. Figure S5: Presence of the interference in the peak Microcystins RR [D-Asp3] + Microcystins RR [D-Asp3, (E)-Dhb7]. Figure S6: The route of the research vessel collecting phytoplankton samples. Table S1: The reference standards of cyanotoxins included in the method. Table S2: MRM transitions in positive ESI mode. Table S3: PRM acquisition in positive ESI mode for the hybrid quadrupole Orbitrap LC-HRMS/MS.
