# Supplementary material for: LC-MS/MS Analysis of Cyanotoxins in Bivalve Mollusks—Method Development, Validation and First Evidence of Occurrence of Nodularin in Mussels (Mytilus edulis) and Oysters (Magallana gigas) from the West Coast of Sweden
Source: Toxins (Basel). 2023 May 11;15(5):329. doi: 10.3390/toxins15050329 (PMC10221150; doi:10.3390/toxins15050329)
Supplement: Supplementary file 1 [file toxins-15-00329-s001.zip › toxins-2346376-supplementary.pdf]

toxins MDPI

Supplementary Materials: LC-MS/MS Analysis of Cyanotoxins in Bivalve Mollusks—Method Development, Validation and First Evidence of Occurrence of Nodularin in Mussels (*Mytilus edulis*) and Oysters (*Magallana gigas*) from the West Coast of Sweden

Julio César España Amórtegui, Heidi Pekar, Mark Dennis Chico Retrato, Malin Persson, Bengt Karlson, Jonas Bergquist and Aida Zuberovic-Muratovic

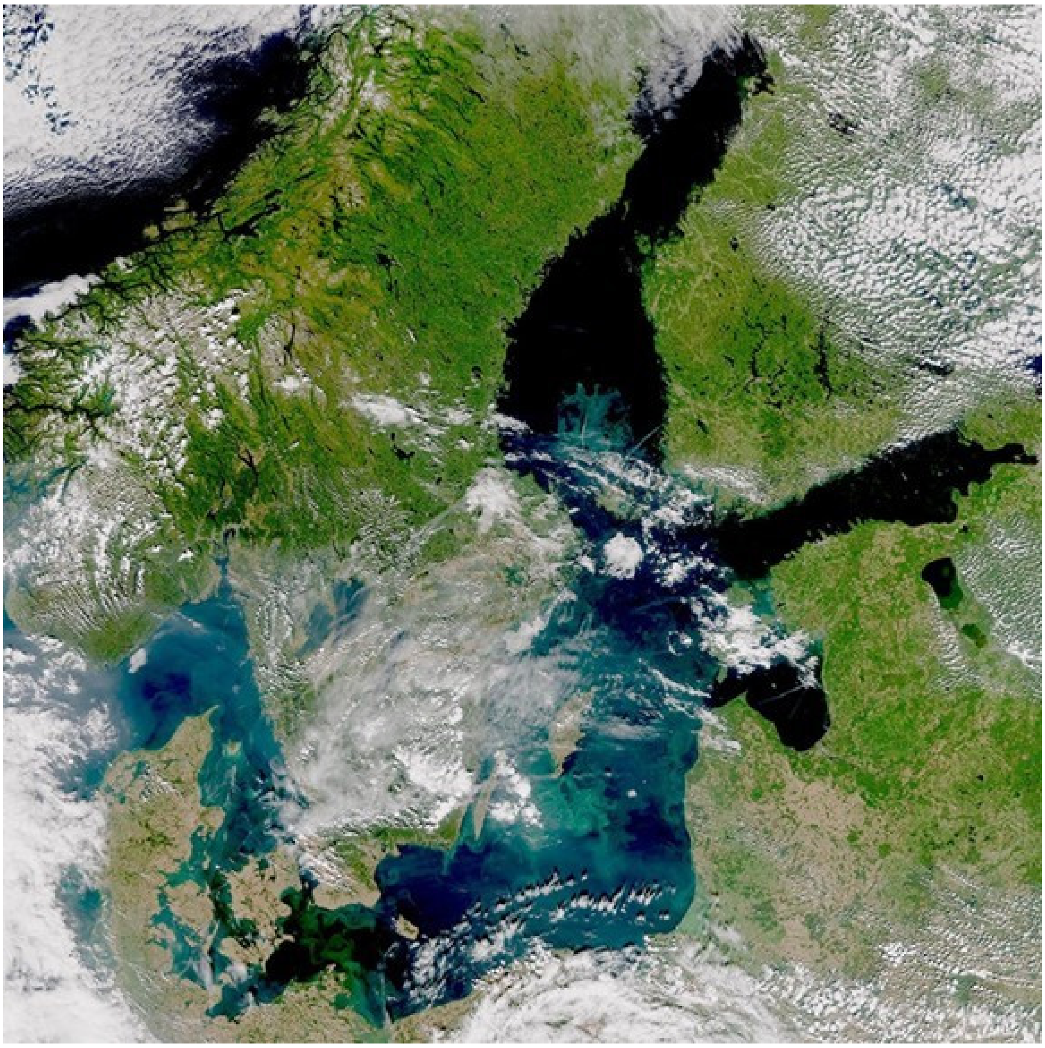

Figure S1. Cyanobacteria bloom, 17 August 2020. Available at the webpage of Swedish Metrological and Hydrologcal Institute (SMHI): https://www.smhi.se/nyhetsarkiv/nystart-for-cyanobakterieblomningen-i-augusti-1.163168.

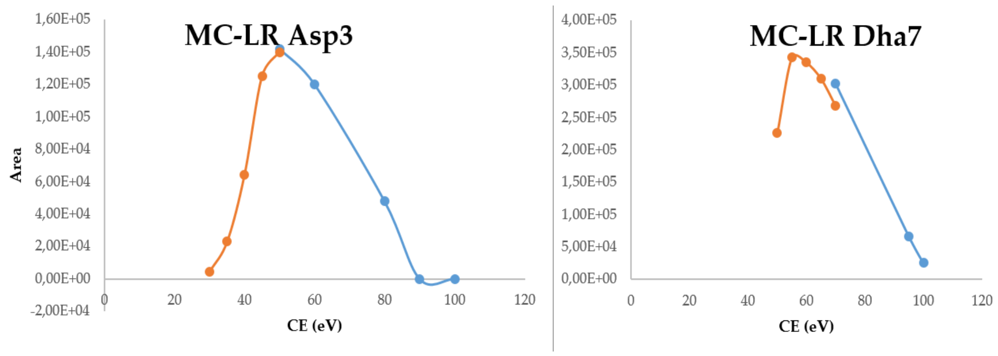

(a) **(b)**

Figure S2. Optimization of the Collision Energy (eV) carried out in two subsequent MRM tests including the ranges in search for an inflexion point. The typical transition 981.5 > 135 was replaced by: (**a**) CE: 55 eV for microcystin LR [Asp3] transition 981.5 > 213; (**b**) CE: 55 eV for microcystin LR [Dha7] transition 981.5 > 269. The colors are different but correspond to the first test ramping down the energy until an arbitrary lower value that was not enough to see the maximum. It was necessary to extend the CE further to the lower bound in order to find an inflexion point.

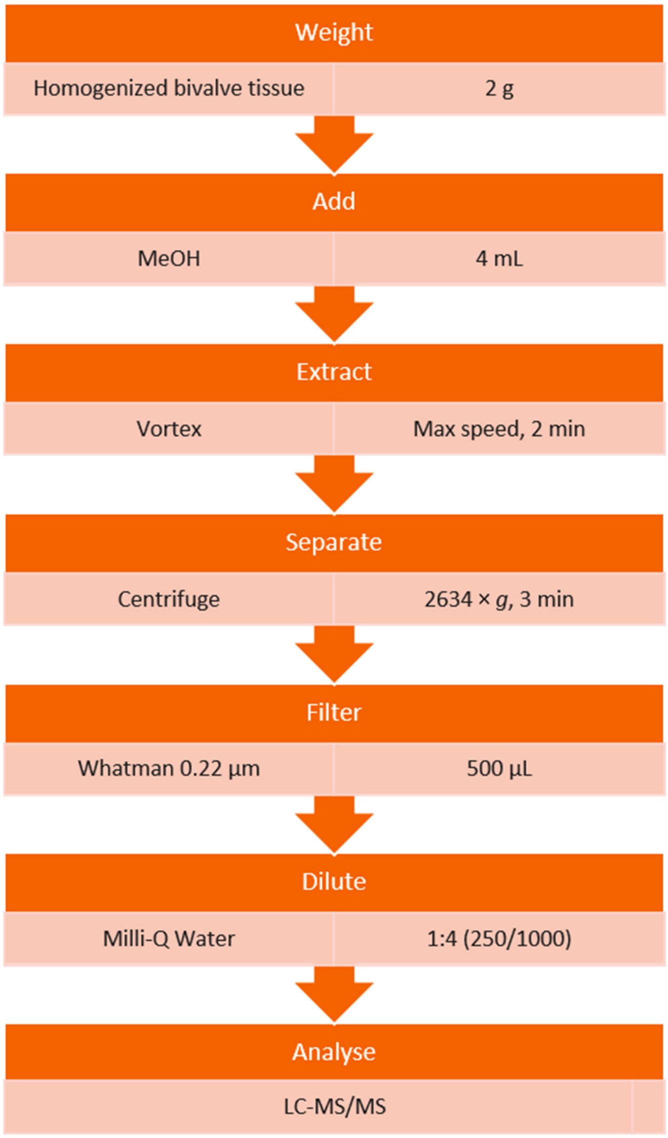

Figure S3. Protocol for the extraction of cyanotoxins from bivalve tissue. The centrifugation speed of 2634 × *g* corresponded to 3500 RPM. **.**

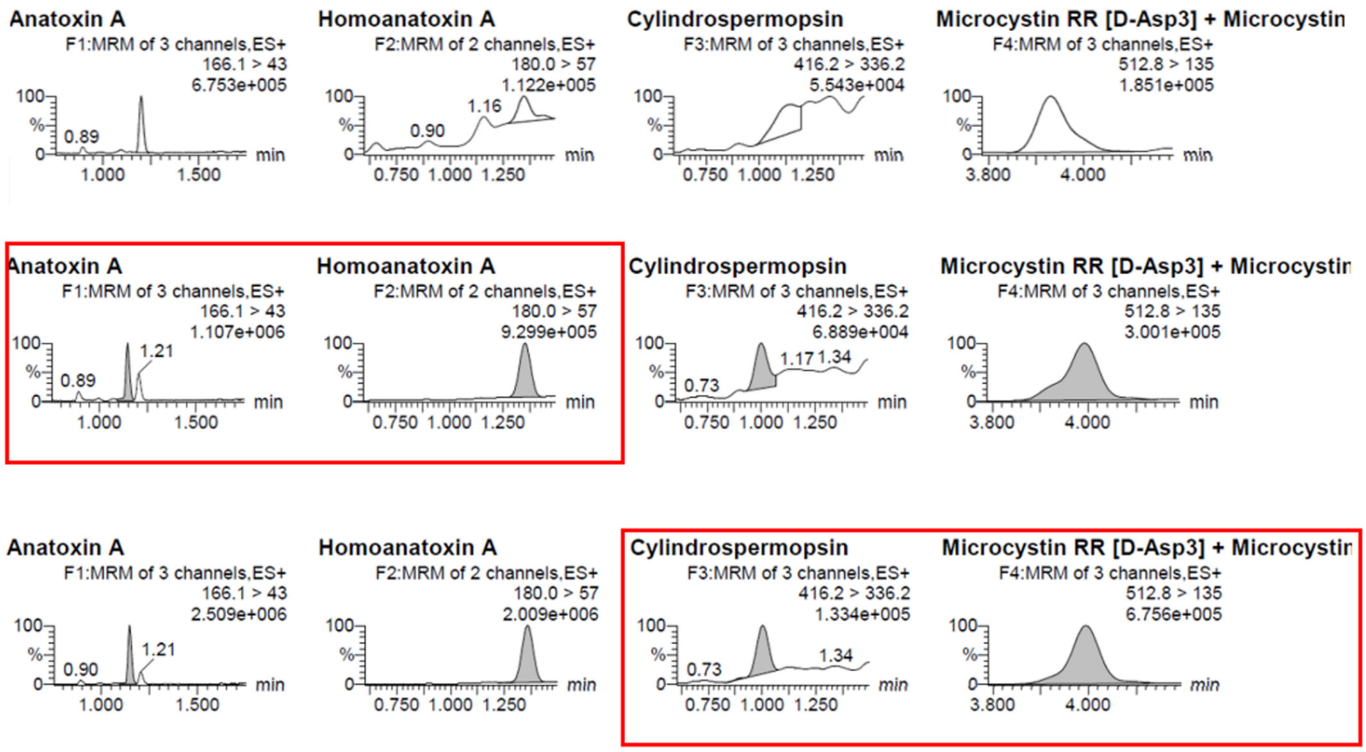

(a) (b) (c) (d)

Figure S4. Selectivity over interferences in mussel matrix, the red square shows the levels chosen as lowest calibration level for each particular toxin. Top to bottom: blank, post-extraction blank with spiked levels L00 and L0, corresponding to 3.13 µg/kg and 6.25 µg/kg, respectively. (**a**) Anatoxin A*:* Adjacent peak with complete chromatographic resolution (**b**) Homonatoxin A: Presence of a peak on the same retention time in blank that is actually small (~10%) compared to the peak spiked at 3.13 µg/kg. (**c**) Cylindrospermopsin: Significant matrix noise resulted in 6.25 µg/kg as the corresponding lowest calibrated level to ensure a higher S/N. (**d**) Microcystins RR [D-Asp3] + RR [D-Asp3, (E)-Dhb7]: The peak prevailed over the interference in blank at 6.25 µg/kg which is only then seen as a small shoulder (<30%).

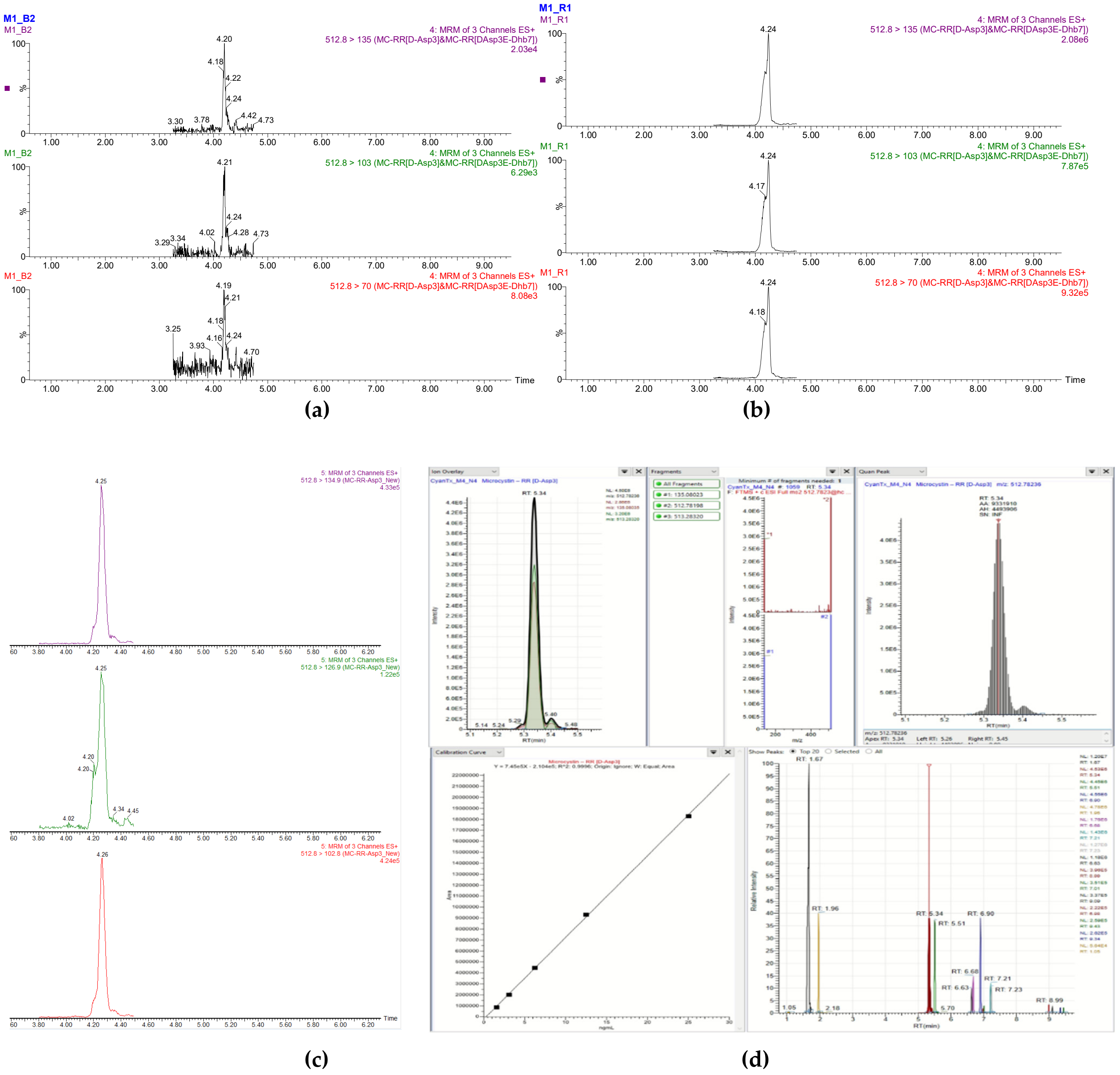

Figure S5. Presence of the interference in the peak Microcystins RR [D-Asp3] + Microcystins RR [D-Asp3, (E)-Dhb7]. **(a)** Peak present in blanks for the quantifying trace and MRM transitions **(b)** The interference adds up a significant area to the toxin peak at a low level (0.38 µg/mL). **(c)** Selected alternative qualifiers candidates still showing the isobaric interference as a shoulder; **(d)** Extracted ion chromatograms of selected ions by orbitrap-based high resolution (17500 FWHM) showing enough data points and excellent linearity down to 1.56 µg/mL.

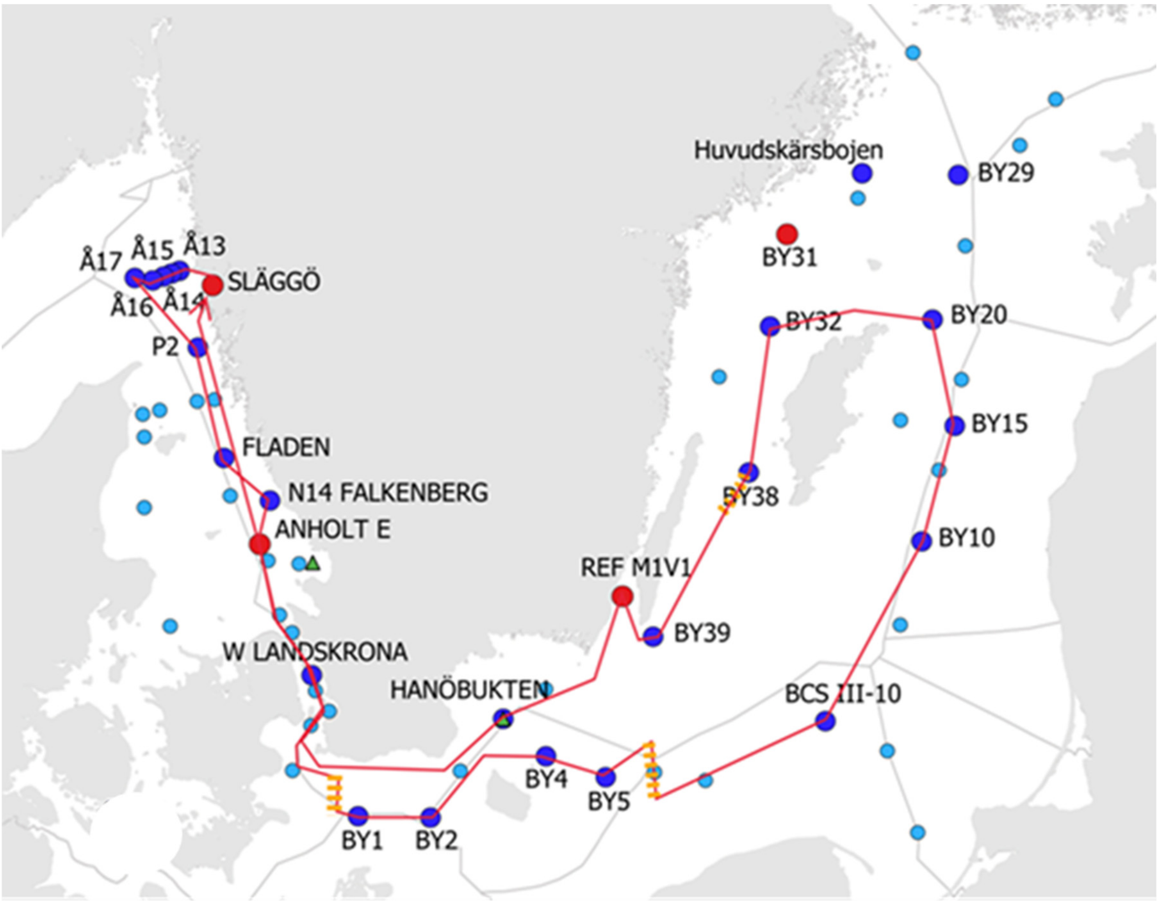

Figure S6. The route of the research vessel collecting phytoplankton samples in southern Sweden in July 2021.

Table S1. The reference standards of cyanotoxins included in the method.

Compound name	Standard mix	Source
Anatoxin-a	A	Teknolab Sverige AB
Homoanatoxin-a	A	Teknolab Sverige AB
Cylindrospermopsin	A	Teknolab Sverige AB
Nodularin	A	Biosense Laboratories A/S
Microcystin-RR	A	AH Diagnostics AB
Microcystin-HtyR	A	AH Diagnostics AB
Microcystin-[D-Asp3]-LR	A	AH Diagnostics AB
Microcystin-LA	B	AH Diagnostics AB
Microcystin-LF	B	Teknolab Sverige AB
Microcystin-LW	B	Teknolab Sverige AB
Microcystin-LY	B	AH Diagnostics AB
Microcystin-YR	B	Teknolab Sverige AB
Microcystin-LR	B	AH Diagnostics AB
Microcystin-WR	C	AH Diagnostics AB
Microcystin-HilR	C	AH Diagnostics AB
Microcystin-[Dha7]-LR	C	Teknolab Sverige AB
Microcystin-[D-Asp3, (E)-Dhb7]-RR	C	Teknolab Sverige AB

Table S2. MRM transitions in positive ESI mode. The transition to quantify the analogues Microcystin LR [Dha7] and Microcystin LR [Asp3] was set at a significantly lower, yet notably more convenient collision energy to achieve selectivity between this isobaric pair. Transitions in bold indicate the quantification trace. *Isobars Microcystins RR [D-Asp3] + Microcystins RR [D-Asp3, (E)-Dhb7].

Toxins	Retention Time (min)	Trace (m/z)	Ref.	Cone (V)	CE (eV)
Anatoxin A	1.15	**166 > 43**	Pekar, 2016	10	20
		166 > 91	Pekar, 2016		20
		166 > 149	Lemoin, 2013		10
Homoanatoxin A	1.35	**180 > 57**	Pekar, 2016	10	20
		180 > 163	Pekar, 2016; Roy–Lachapelle, 2019		10
Cylindrospermopsin	1.03	416 > 176	Pekar, 2016	40	40
		416 > 194	Pekar, 2016; Roy–Lachapelle, 2019		30
		**416 > 336**	Pekar, 2016; Roy–Lachapelle, 2019		20
*Microcystins RR Sum**	3.99	513 > 70	Pekar, 2016	60	50
		513 > 103	Pekar, 2016		50
		**513 > 135**	Pekar, 2016		30
Microcystin RR	4.25	520 > 103	Pekar, 2016; Turner, 2018	30	70
		520 > 127	Pekar, 2016; Turner, 2018		50
		**520 > 135**	Pekar, 2016; Turner, 2018		30
Nodularin	5.07	825.5 > 103	Pekar, 2016; Turner, 2018	55	100
		**825.5 > 135**	Pekar, 2016; Turner, 2018		60
Microcystin LA	7.95	910 > 107	Turner, 2018	35	80
		**910 > 135**	Pekar, 2016; Turner, 2018		70
		910 > 375	Birbeck, 2019		70
		910 > 776	Pekar, 2016; Roy–Lachapelle, 2019		80
Microcystin LR [Dha7]	5.85	981.5 > 113	----	75	50
		981.5 > 135	Pekar, 2016; Turner, 2018		70
		**981.5 > 269**	----		50
Microcystin LR [Asp3]	6.02	981.5 > 127	----	75	60
		981.5 > 135	Pekar, 2016; Turner, 2018		75
		**981.5 > 213**	----		60
Microcystin LF	8.90	**986.5 > 135**	Pekar, 2016; Turner, 2018	35	65
		986.5 > 213	Turner, 2018		60
		986.5 > 478	Pekar, 2016; Birbeck, 2019		40
		986.5 > 852	Birbeck, 2019		40
Microcystin LR	5.89	995.6 > 127	Turner, 2018; Birbeck, 2019	60	90
		**995.6 > 135**	Pekar, 2016; Turner, 2018		70
Microcystin LY	8.11	1002.5 > 107	Pekar, 2016; Turner, 2018	40	90
		**1002.5 > 135**	Pekar, 2016; Turner, 2018		70
		1002.5 > 494	Birbeck, 2019		50
		1002.5 > 868	Pekar, 2016		50
Microcystin HilR	6.22	1009.7 > 107	Turner, 2018	75	90
		1009.7 > 127	Turner, 2018		80
		**1009.7 > 135**	Pekar, 2016		75
Microcystin LW	8.81	1025.5 > 127	Turner, 2018	35	90
		**1025.5 > 135**	Pekar, 2016; Turner, 2018		65
		1025.5 > 517	Birbeck, 2019, Roy–Lachapelle, 2019		35
Microcystin YR	5.50	**1045.6 > 127**	Turner, 2018	75	90
		1045.6 > 135	Pekar, 2016; Turner, 2018		75
Microcystin HtyR	5.64	**1059.6 > 135**	Pekar, 2016; Turner, 2018	75	90
		1059.6 > 107	Pekar, 2016; Turner, 2018		70
Microcystin WR	6.23	**1068.6 > 135**	Pekar, 2016; Turner, 2018	80	100
		1068.6 > 107	Pekar, 2016; Turner, 2018		75

Table S3. PRM acquisition in positive ESI mode for the hybrid quadrupole-orbitrap LC-HRMS/MS. *CE: Collision energy at the HCD (Higher Energy Collision Induced Dissociation), MS/MS stage able to produce triple quadrupole-like product ion mass spectra. *Isobaric species of Microcystins RR [D-Asp3] + Microcystins RR [D-Asp3, (E)-Dhb7].

Toxin name	Retention Time (min)	Precursor (m/z)	Charge (z)	*CE (eV)	Product ions mass range (m/z)
Anatoxin-a	1.66	166.1226	+1	10	50-190
Homoanatoxin-a	1.95	180.1383	+1	25	50-205
Cylindrospermopsin	1.08	416.1234	+1	25	50-445
*Microcystin – RR Sum**	5.37	512.7823	+2	25	71.3-1070
Microcystin – RR	5.52	519.7902	+2	25	72.3-1085
Microcystin – LA	8.92	776.4189	+1	50	54-810
Nodularin – NOD	6.10	825.4505	+1	40	57.3-860
Microcystin – LR [D-Asp3] & LR [Dha7]	6.95	981.5404	+1	65	68-1020
Microcystin – LF	9.40	986.5234	+1	50	68.3-1025
Microcystin – LR	6.87	995.5560	+1	25	69-1035
Microcystin – LY	9.06	1002.5343	+1	50	69.7-1045
Microcystin – HilR	7.19	1009.5717	+1	50	70-1050
Microcystin – LW	9.32	1025.5343	+1	50	71-1065
Microcystin – YR	6.60	1045.5353	+1	50	72.3-1085
Microcystin – HtyR	6.66	1059.5509	+1	50	73.3-1100
Microcystin – WR	7.21	1068.5513	+1	50	74-1110
